# Demographical and Clinical Factors Predictive for Aortic Dilatation. When should we be Concerned about the Size?

**DOI:** 10.31083/j.rcm2505150

**Published:** 2024-04-30

**Authors:** Tomasz Urbanowicz, Justyna Rajewska-Tabor, Anna Olasińska-Wiśniewska, Krzysztof J. Filipiak, Michał Michalak, Patrycja Rzesoś, Mateusz Szot, Aleksandra Krasińska-Płachta, Beata Krasińska, Małgorzata Pyda, Andrzej Tykarski, Marek Jemielity, Zbigniew Krasiński

**Affiliations:** ^1^Cardiac Surgery and Transplantology Department, Poznan University of Medical Sciences, 61-848 Poznań, Poland; ^2^Unit of Magnetic Resonance, I Department of Cardiology, Poznan University of Medical Sciences, 61-848 Poznań, Poland; ^3^Department of Hypertensiology, Angiology and Internal Medicine, Poznan University of Medical Sciences, 61-848 Poznań, Poland; ^4^Institute of Clinical Science, Maria Sklodowska-Curie Medical Academy, 60-806 Warsaw, Poland; ^5^Department of Computer Science and Statistics, Poznan University of Medical Sciences, 61-107 Poznań, Poland; ^6^Medical Faculty, Poznan University of Medical Sciences, 61-107 Poznań, Poland; ^7^Department of Ophthalmology, Poznan University of Medical Sciences, 60–569 Poznań, Poland; ^8^Department of Vascular, Endovascular Surgery, Angiology and Phlebology Medical University, Poznan University of Medical Science, 61-848 Poznań, Poland

**Keywords:** aortic dilatation, thoracic aorta, abdominal aorta, MRI

## Abstract

**Background:**

Thoracic aortic aneurysms are often an accidental finding and result from a degenerative process. Medical therapy includes pharmacological control of arterial hypertension and smoking cessation, that slows the growth of aneurysms. An association between the dilatation of the ascending and abdominal aorta has been already reported. The aim of the study was to identify possible demographic and clinical factors that may implicate further imaging diagnostics in patients with ascending aorta dilatation.

**Methods:**

There were 181 (93 (53%) males and 88 (47%) females) patients with a median age of 54 (41–62) years who underwent cardiac magnetic resonance due to non-vascular diseases, were enrolled into retrospective analysis.

**Results:**

Multivariable analysis revealed ascending aorta dilatation (odds ratios (OR) = 7.45, 95% confidence interval (CI): 1.98–28.0, *p* = 0.003) and co-existence of coronary artery disease (OR = 8.68, 95% CI: 2.15–35.1, *p* = 0.002) as significant predictors for thoracic descending aorta dilatation. In patients with abdominal aorta dilatation, the multivariable analysis showed a predictive value of ascending aortic dilatation (OR = 14.8, 95% CI: 2.36–92.8, *p* = 0.004) and age (OR = 1.04, 95% CI: 1.00–1.08, *p* = 0.027). In addition, cut-off values were established for age groups determining the risk of thoracic aorta dilatation over 49 years and abdominal aorta dilatation over 54 years.

**Conclusions:**

The results of our analysis showed predictive factors, including ascending aorta dilatation and co-existence of coronary artery disease, particularly over 49 years of age for thoracic, while ascending aorta dilatation and age, particularly over 54 years, for abdominal aorta dilatation. These features may be considered to increase clinical vigilance in patients with aortic diameter abnormalities.

## 1. Introduction

Thoracic aortic aneurysms are often an accidental finding and result from a degenerative process [1]. Medical therapy involves pharmacological control of arterial hypertension and smoking cessation that slows the growth of aneurysms [2, 3]. Aortic dissection is a life-threatening condition that, in more than 50% of cases, is located in previously dilated, and not aneurysmal locations [4]. It is reported as a causative factor of over 25,000 deaths annually in the United States [5]. Guo *et al*. [6] reported a mean ascending aortic diameter related to acute dissection of 42.6 mm. The interaction of molecular proteins with the cellular and intercellular matrix is claimed to be related to the progression of aortic diameter [7, 8, 9].

The diagnosis of aortic pathology is based on imaging examinations, including ultrasonography followed by computed tomography (CT) or magnetic resonance angiography (MRA) [10, 11]. Ultrasound imaging can be helpful only in certain segments of the aorta, including echocardiography of the ascending aorta [12]. The rest of the aorta is only partially visible or invisible at all. Noninvasive imaging are often the first-line test that allow for the suggestion of further diagnostics including MRA or CT scans [13, 14]. Considerations regarding improved assessment of the risk of dissection and rupture based on imaging modalities are still controversial [15, 16]. MRA and CT are valuable in the evaluation of chronic dissection, while CT is dedicated to acute syndromes. The advantages of MRA include the lack of radiation and the use of gadolinium contrast media with low risk of complications, which allows for repeatable examinations and safe utility in the younger population.

Screening programs are believed to be one of the most effective options for diagnosing aortic aneurysms, as incidental findings of aortic dilatation are most common [17, 18]. The increased risk of multiple dilated segments in a single patient is well-known [19].

The aim of the study was to identify possible demographic and clinical factors that may implicate further imaging diagnostics in patients with ascending aorta dilatation.

## 2. Materials and Methods

### 2.1 Group Characterization

One hundred and eighty-one consecutive patients who underwent cardiac magnetic resonance (CMR) for non-vascular diseases were included in the analysis. The main primary indications were examination of patients with suspected pulmonary hypertension in 61 (34%) cases, analysis before pulmonary veins isolation in atrial fibrillation in 50 (28%) subjects and other reasons in 70 (39%) patients. All patients underwent MRA during CMR. Exclusion criteria included previously diagnosed aortic aneurysms and dissections (chronic or acute) and inability to perform the examination.

Four aortic diameters were measured in the ascending aorta above the sinotubular (ST) junction, the descending aorta 1 cm below the origin of the left subclavian artery, the descending aorta at the level of the diaphragm and the abdominal aorta 2 centimeters above the bifurcation into two common iliac arteries.

Demographic and clinical data were collected based on a questionnaire completed by each patient.

### 2.2 Magnetic Resonance Imaging

All the patients had CMR for non-vascular reasons. CMR was performed on a 1.5 T scanner Magnetom Avanto Fit (Siemens, Erlangen, Germany). CMR and 3-dimensional (3D) dynamic magnetic resonance (MR) angiography were performed with a 1.5-T scanner (Siemens, Avanto Fit) with the use of a matrix coil for body and cardiac applications combined with a spinal coil. All sequences were performed with electrocardiographic (ECG) triggering during breath-hold. CMR included the following sequences: anatomical imaging, ventricular volume and functional assessment and phase-contrast flow quantification. MRA was performed with the use of a dynamic Time-resolved Angiography With Interleaved Stochastic Trajectories (TWIST) after the administration of a contrast agent gadobutrol (0.1 mmol/kg) followed immediately by a 20 mL saline flush. The temporal resolution varied between 3 and 5 s, with an overall sequence time of ~100 s. The time of contrast injection was calculated following the administration of 2 mL of contrast bolus. The typical sequence parameters were: repetition time/echo time (TR/TE) 2.3/0.87 ms, field of view 500 × 310 mm, slice thickness 1.5 mm, gap 0 mm, matrix size 384 × 224, and in-plane resolution 1.40 × 1.30 mm. The TWIST sequence was used for the evaluation of vascular anomalies and the following measurements. The aortic diameter was measured at four levels including the ascending aorta (A1), descending thoracic aorta 1 centimeter below left subclavian artery origin (A2), abdominal aorta at the level of diaphragm (A3), and abdominal aorta 2 centimeters above bifurcation into two ileac arteries (A4). MRA images evaluation was based on criteria proposed by van Hout *et al*. [20] in segments in risk for aortic dilatation [3]. All measurements were obtained with dedicated software Horos.

The A1 segment dilatation and aneurysm were considered when the transverse diameter was greater than 3.7 and 5 cm, respectively [21]. For each aortic segment, the aneurysms were defined as 1.5 times the normal value [22]. The A2 abnormal diameter was defined as exceeding 2.9 cm [23]. In A3 and A4 aortic segments, the transverse diameters above 2.5 cm and 1.9 cm were taken into analysis as enlarged, respectively [24, 25].

The diameter of each segment was manually calculated by 2 measurements that were performed in transverse planes perpendicular to the aortic axis by 2 operators blinded to the other measurements and clinical features. Mean values of diameters of the aorta performed by both operators were included in the analysis.

### 2.3 Statistical Analysis

Since numerical data did not follow a normal distribution (Shapiro–Wilk test) the results were presented as medians and interquartile ranges — median ($Q_{1}$–$Q_{3}$). The categorical data were presented as numbers and relative frequencies. Univariable and multivariable logistic regression with backward stepwise selection was used to find significant predictors of thoracic and abdominal aorta — segments A2 and A3 and A4 — dilatations. The results were presented as odds ratios (OR) and 95% confidence intervals (95% CI). Additionally, the receiver operating characteristic curve (ROC) analysis was performed to determine the classifier’s discriminatory power and to identify the optimal cut-off point of age (Youden index method) for thoracic and abdominal aorta dilatation. The discriminatory power was measured as the area under the curve (AUC). A *p* value < 0.05 was considered statistically significant.

### 2.4 Ethics Approval and Consent to Participate

The study was performed according to the principles of Good Clinical Practice and the Declaration of Helsinki and was approved by the Local Ethics Committee of the Medical University of Poznan (approval number: 749/20 on 4 November 2020). All patients gave their informed consent for inclusion in the analysis.

## 3. Results

There were 181 (93 (53%) males and 88 (47%) females) patients with a median age of 54 (41–62) years enrolled into the retrospective study. Two risk factors, most common for aneurysms’ occurrence, namely arterial hypertension and smoking, were diagnosed in 92 (51%) and 65 (36%) patients, respectively. The physically active lifestyle was postulated by 50 (28%) patients and was characterized by a median frequency of activity 3 (1–4) times a week. The demographic and clinical data were analyzed and presented in Table 1.

**Table 1.** **Demographical and clinical data**.

		n = 181
Demographical:		
	1. Age (years median (Q1–Q3))	54 (41–62)
	2. Sex (male (%)/female (%))	93 (53%)/88 (47%)
	3. Height (cm) (median (Q1–Q3))	84 (70–96)
	4. Weight (kg) (median (Q1–Q3))	172 (164–1820)
Comorbidities:		
	1. Arterial hypertension (n (%))	92 (51)
	2. Hyperlipidemia (n (%))	74 (41)
	3. Coronary artery disease (n (%))	25 (14)
	4. Diabetes mellitus (n (%))	15 (8)
	5. Peripheral artery disease (n (%))	32 (18)
	6. Chronic obstructive pulmonary disease (n (%))	27 (15)
	7. Family history (n (%))	92 (51)
Smoking overall (%):		65 (36)
	1. Current (n (%))	23 (13)
	2. Past smoking (n (%))	42 (23)
Lifestyle:		
	1. Active physically (n (%))/sedentary (n (%))	50 (28)/131 (72)
	2. Activities a week (median (Q1–Q3))	3 (1–4)
Pharmacology:		
	1. B-blockers (n (%))	73 (40)
	2. ACE-I (n (%))	68 (38)
	3. Ca-blockers (n (%))	34 (19)
	4. Anti-platelet (n (%))	20 (11)
	5. NOAC (n (%))	52 (29)
	6. Statins (n (%))	52 (29)
	7. Diuretics (n (%))	48 (27)
	8. Other (n (%))	34 (19)

Abbreviations: ACE-I, angiotensin-converting enzyme inhibitor; B, beta receptor; Ca, calcium receptor; n, number; NOAC, non-vitamin K antagonist oral anticoagulants.

**Fig. 1.**
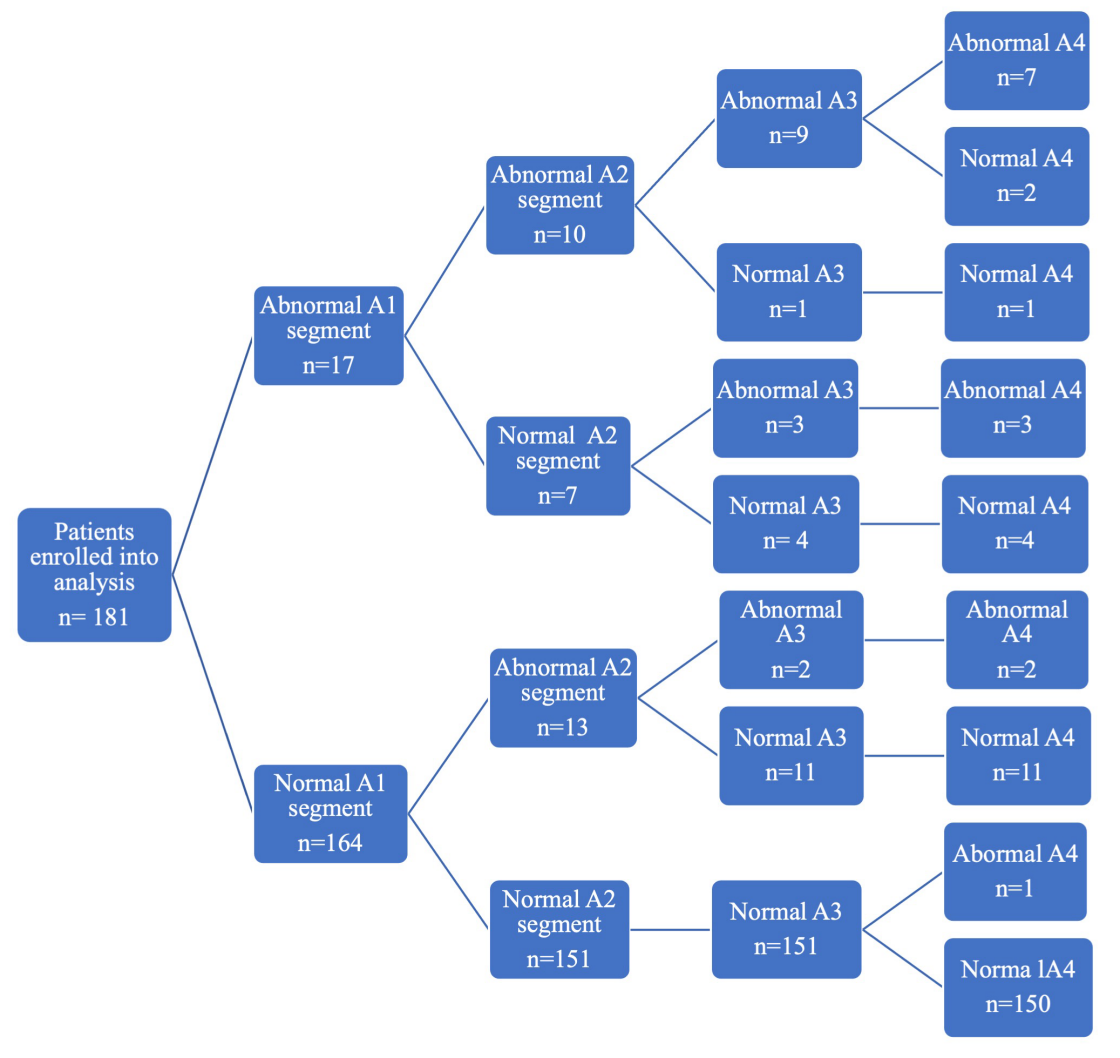
**Flow chart presenting the MRI results in A11-A4 aortic segments**. MRI, magnetic resonance imaging.

Data obtained from the MRA results revealed that median (Q1–Q3) values of four analyzed aortic diameters, including ascending aorta (A1) as 28 (19–48) mm, descending aorta below subclavian artery origin (A2) — 24 (21–40) mm, thoracic aorta below the diaphragm level (A3) — 21 (15–28) mm and above aortic division into common iliac arteries (A4) — 16 (12–20) mm. The Fig. 1 presents the normal and abnormal (dilatation and aneurysms) aortic diameters in A1-A4 segments.

The diagnostic criteria for aortic aneurysm were met in 4 (2%) patients in ascending aorta. The results are presented in Table 2. The A2–A4 diameters were analyzed in relation to the diameters of A1. The risk for A2–A4 dilatation was evaluated by uni- and multivariable analysis in patients with an A1 diameter above the normal range. The analysis was performed for each of A2–A4 diameters.

**Table 2.** **Median values of aortic diameters from MRI analysis**.

Parameters		n = 181
Ascending aorta (A1 segment)		
	1. median value (mm) (Q1–Q3)	28 (19–48)
	2. enlargement (n (%))	15 (8)
	3. aneurysm diagnosis (n (%))	2 (1)
Descending aorta (A2 segment) thoracic		
	1. median value (mm) (Q1–Q3)	24 (17–41)
	2. enlargement (n (%))	17 (10)
	3. aneurysm diagnosis (n (%))	3 (2)
Descending aorta (A3 segment) below diaphragm		
	1. median value (mm) (Q1–Q3)	22 (15–39)
	2. enlargement (n (%))	11 (6)
	3. aneurysm diagnosis (n (%))	3 (2)
Descending aorta (A4 segment) abdominal		
	1. median value (mm) (Q1–Q3)	17 (14–22)
	2. enlargement (n (%))	13 (7)
	3. aneurysm diagnosis (n (%))	0

Abbreviations: A, aortic; MRI, magnetic resonance imaging; n, number; Q, quartiles.

### 3.1 Descending Thoracic Aorta (A2)

Univariable analysis found a significant relation between ascending aorta dilatation and A2 diameter (OR = 5.86, 95% confidence interval (CI): 1.04–32.8, *p* = 0.045), age (OR = 1.07, 95% CI: 1.02–1.13, *p* = 0.011), followed by the co-existence of coronary artery disease (OR = 7.45, 95% CI: 1.98–28.0, *p* = 0.003).

Multivariable analysis found a significant relation between ascending aorta dilatation and A2 diameter (OR = 8.0, 95% CI: 1.21–53.1, *p* = 0.031) and the co-existence of coronary artery disease (OR = 8.68, 95% CI: 2.15–35.1, *p* = 0.002) as presented in Table 3.

**Table 3.** **Uni- and multivariable analysis for A2 and A3 aortic segment dilatation prediction**.

			OR	95% CI	*p*
Thoracic aorta	Univariable				
Segment A2		1. ascending aorta dilatation	5.86	1.04–32.8	0.045
		2. co-existence of coronary artery disease	8.00	1.21–53.1	0.031
	Multivariable				
		1. ascending aorta dilatation	7.45	1.98–28.0	0.003
		2. co-existence of coronary artery disease	8.68	2.15–35.1	0.002
Thoracic aorta	Univariable				
Segment A3		1. ascending aorta dilatation	8.00	4.04–104.8	<0.001
		2. female sex	0.41	0.18–0.93	0.034
		3. co-existence of arterial hypertension	2.45	1.09–5.53	0.031
		4. co-existence of coronary artery disease	3.20	1.27–8.11	0.014
	Multivariable				
		1. ascending aorta dilatation	14.8	2.36–92.8	0.004
		2. age	1.04	1.00–1.08	0.027

Abbreviations: A2 segment, descending aorta 1 cm below subclavian artery origin; A3 segment, descending aorta 1 cm below diaphragm level; CI, confidence interval; OR, odds ratios.

### 3.2 Aortic Diameter on the Diaphragmatic Level (A3)

Univariable analysis found a significant correlation between ascending aorta dilatation and A3 diameter (OR = 20.58, 95% CI: 4.04–104.8, *p* < 0.001). Moreover, the female sex (OR = 0.41, 95% CI: 0.18–0.93, *p* = 0.034) followed by the co-existence of arterial hypertension (OR = 2.45, 95% CI: 1.09–5.53, *p* = 0.031) and coronary artery disease (OR = 3.20, 95% CI: 1.27–8.11, *p* = 0.014) were found significant.

Multivariable analysis found a significant relation between ascending aorta dilatation and A3 diameter (OR = 14.8, 95% CI: 2.36–92.8, *p* = 0.004) followed by age (OR = 1.04, 95% CI: 1.00–1.08, *p* = 0.027) as presented in Table 3.

### 3.3 Aortic Diameter above Division (A4) 

In univariable and multivariable analysis, there was no relationship between A1 and A4 diameters.

### 3.4 Receiver Operator Curve Analysis

#### 3.4.1 ROC Curve Analysis for Descending Aorta Dilatation Related to Ascending Aorta Dilatation

**Fig. 2.**
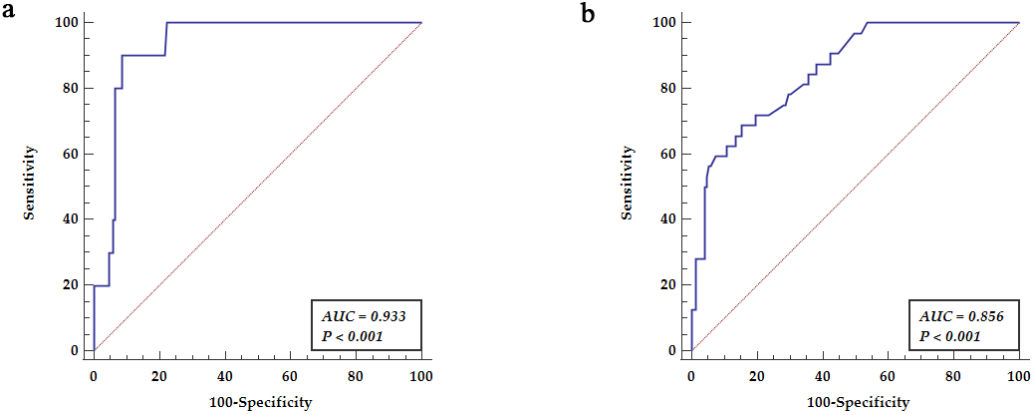
**Receiver operator curve analysis for non-ascending aorta diameter relation to ascending aorta**. (a) Receiver operator curve analysis for aortic A2 segments dilatation related to the diameter of the ascending aorta (segment A1). (b) Receiver operator curve revealed predictive values for abdominal aorta dilatation (segment A3) related to the diameter of the ascending aorta (segment A1). AUC, area under the curve.

ROC analysis for aortic A2 segments dilatation related to the diameter of the ascending aorta (segment A1) revealed the area under the curve (AUC = 0.933, *p* < 0.001) yielded sensitivity of 90% and specificity of 91.23% as presented in Fig. 2a. ROC analysis revealed predictive values for abdominal aorta dilatation (segment A3) related to the diameter of the ascending aorta (segment A1) (AUC = 0.856, *p* < 0.001) yielding sensitivity of 68.75% and specificity of 84.56% as presented in Fig. 2b.

#### 3.4.2 Receiver Operator Curve Analysis for Aortic Dilatation Related to Age

**Fig. 3.**
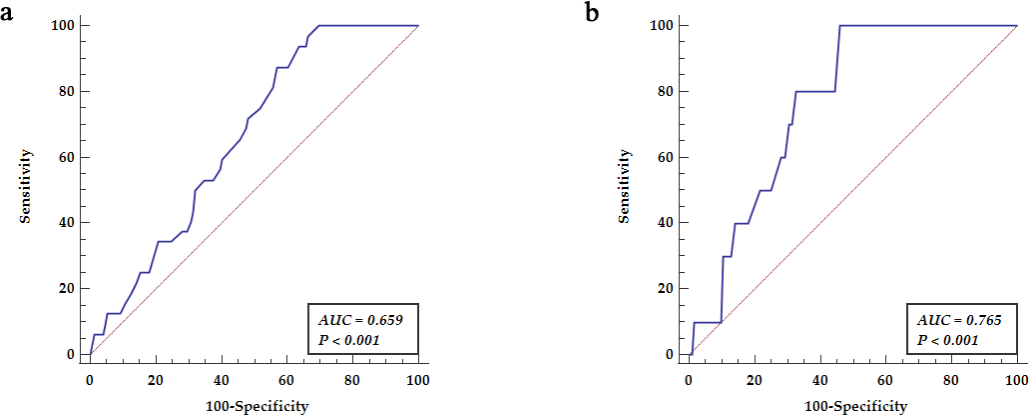
**Receiver operator curve analysis relating age to aortic dilatation**. (a) Receiver operator curve analysis revealed predictive values for thoracic aorta dilatation (segment A2) related to the age. (b) Receiver operator curve analysis revealed predictive values for abdominal aorta dilatation (segment A3) related to the age. AUC, area under the curve.

ROC analysis revealed predictive values for thoracic aorta dilatation (segment A2) related to the age, representing the area under the curve (AUC = 0.659, *p* < 0.001) with an optimal cut off value over 49 years, yielding sensitivity of 87.50% and specificity of 42.95% as presented in Fig. 3a. ROC analysis revealed predictive values for abdominal aorta dilatation (segment A3) related to the age, presenting area under the curve (AUC = 0.765, *p* < 0.001) with an optimal cut off value above 54 years, yielding a sensitivity of 100% and a specificity of 53.80% as presented in Fig. 3b.

#### 3.4.3 ROC Curve Analysis for Descending Aorta Dilatation in the Multivariable Analysis

**Fig. 4.**
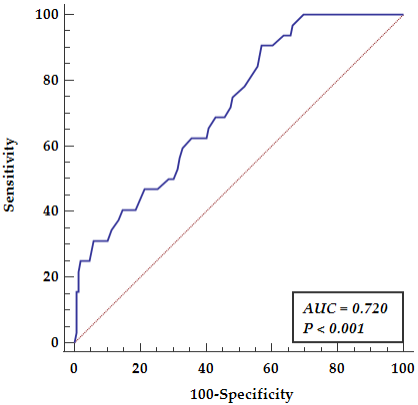
**Receiver operator curve analysis presenting multifactorial predictive score for aortic dilatation in A2 and A3 segments**. AUC, area under the curve.

ROC curve analysis for aortic enlargement in A2 and A3 segments related to the multifactorial predictive score constructed based on age over 50 years, ascending aorta dilatation over 36 mm and co-existence of coronary artery disease revealed the area under the curve (AUC = 0.720, *p* < 0.001), yielding a sensitivity of 90.62% and a specificity of 42.95% as presented in Fig. 4.

## 4. Discussion

According to the results of the study, patients with ascending aorta dilatation who are over 49 years of age and with concomitant coronary artery disease (CAD) are more predisposed to dilatation of thoracic aorta dilatation and those over 54 years of age are more predisposed to abdominal aorta dilatation. The predictive value of these three parameters for thoracic and abdominal aorta dilatation was presented in our logarithmic regression predictive score and may be applied in clinical practice for screening tests.

In western countries, the prevalence of aortic aneurysms is reported in 1–2% of women and 1–9% of men [26]. Screening for aneurysms is fundamental to avoid acute aortic syndromes, which are related to high death rates [4].

In the thoracic segments of the aorta, 60% of aneurysms are reported in the ascending part [27]. Arterial hypertension, family history, smoking habits, and male sex are reported as well-known risk factors for aortic aneurysm development [28]. In patients presenting with increased cardiovascular risks, the US Preventive Services Task Force has endorsed screening ultrasound tests for abdominal aneurysms [29]. In the meta-analysis by Kobeissi *et al*. [30] hypertension increases the risk of developing abdominal aortic aneurysm (AAA) by 66%. Vascular wall changes, impaired endothelial cell function and aortic elasticity are included in the pathogenesis of arterial hypertension and aortic aneurysm, which may suggest that mechanisms are closely related [31]. The effect of smoking on the risk of aneurysm occurred higher than on the risk of coronary artery disease or peripheral vascular disease [32]. Data on the influence of diabetes are conflicting, since several studies did not prove a negative effect of hyperglycemia on aneurysm occurrence and even suggested a beneficial aspect of diabetes [33, 34]. This controversial phenomenon was explained by collagen and elastin alterations within the wall of the aorta leading to inhibited secretion of metalloproteinases and prevention of excessive proteolysis, improvement of wall stability and reduced rate of expansion; moreover, lifestyle changes in diabetic patients introduced to reduce blood glycaemia were supposed as beneficial against aortic aneurysm progression [35]. Interestingly, diabetes was not revealed as associated with either thoracic or abdominal aortic dilatation. On the contrary, obesity was associated with aortic aneurysm development, concerning the release of adipokines and induction of aortic inflammation, which may lead to vessel weakening and subsequent wall abnormalities [35]. Moreover, co-morbidities and risk factors in obese patients are similar to the aforementioned ones.

Our presented analysis indicates that patients with ascending aorta dilatation, which can be easily assessed in the screening echocardiography, should be considered for further examination of dilatation of the other aortic segments, particularly in the coexistence of coronary artery disease.

Our study confirms previous reports including Ballegaard *et al*. [36] and Hernesniemi *et al*. [37] analyses, which claimed the consistently high prevalence of coronary artery disease and coexistence of abdominal aneurysms in the current population. However, an Italian group [38] did not confirm a relationship between abdominal aneurysms and atherosclerotic changes in other arterial districts. Kim* et al*. [39] proved that the prevalence of carotid artery disease is significantly higher in patients with abdominal aneurysms than in patients with thoracic aneurysms. We analyzed patients without a known aortic aneurysm and showed that even aortic dilatation not reaching the criteria of aneurysm may be associated with the co-existence of coronary artery disease. The novelty of our analysis is supported by the relevance of the finding that coronary disease coexistence is associated with not only abdominal aortic dilatation presented by segment A3 diameters, but also thoracic aorta diameter dilatation. In our analysis, diameter was also relevant for thoracic aorta dilatation and was presented as a novel valuable factor for descending aorta dilatation.

Our analysis did not focus on aortic aneurysms but an increased aortic diameter should promote scrutiny and close inspection for those patients who are at risk for aneurysm development. In our opinion, finding that the dilatation of ascending aorta does not fit the criteria of an aneurysm may unduly reassure the safety of the patient and therefore delay further diagnostics. In fact, dilatation of the ascending aorta, particularly in patients over 50 years of age and concomitant coronary artery should warrant broadening of the diagnostics to evaluate diameters of the descending aorta.

The analysis was based on MRA results of patients with non-vascular diseases, and the criteria for aortic dilatation were based on previous reports including 36 mm for ascending aorta [40], 30 mm for descending aorta below subclavian artery origin [41], 24 mm for descending aorta below diaphragm [42], and 20 mm for aorta above division into common iliac arteries [43]. Importantly, our study group was composed of patients with no previous diagnosis of aortic dilatation, in whom the exam was planned for other reasons, while our results indicate that the incidental findings may justify the need for screening tests.

The early recognition of aortic diameter enlargement should lead to the intensification of antihypertensive pharmacotherapy, which is believed to influence the progression of aortic dilatation [1, 2]. The body mass index (BMI) reduction combined with appropriate pharmacotherapy directed on cardiovascular risk modification can help reduce the aortic dilatation rate [44]. Aortic aneurysm is characterized by active processes, including local inflammatory activation [6, 8, 45]. Endothelial dysfunction as an early pathologic event in aneurysm formation is claimed to contribute to either oxidative stress or inflammatory degeneration of the arterial wall [46]. Endothelial dysfunction is accompanied by the inability of aortic wall smooth muscle cells to generate force through the elastin-contractile units in response to pulsatile blood flow [7, 47]. Current pharmacotherapy includes a wide range of medications, including antihypertensive and antidiabetic [48, 49]. The potential of anti-inflammatory agents and antibiotic therapy requires large, randomized studies to be regarded as a therapeutic option, as the effective pharmacotherapy of aortic dilatation is still lacking [50].

In the majority of people, aortic aneurysms are incidental findings [51], which indicates the need for proper screening, as the mortality rate in acute aortic syndromes is still high [52]. In routine screening ultrasound tests, such as echocardiography, the ascending aorta is visualized [53, 54] and its potential dilatation may be regarded as a potential indicator for further evaluation.

The novelty of our study is based on identifying the relationship between ascending aorta and abdominal and thoracic aorta dilatation. Until now, a relationship between aneurysms of ascending and abdominal segments had only been postulated in the Gallego-Colon *et al*. study [55].

The study was designed based on CMR examinations in a population without a previous diagnosis of aortic dilatation. The screening tests should consider not only the MRA, but also computed tomography angiography due to its wider availability. However, the MRA can not only reveal the dilatation of the aorta but is also used to determine pulse wave velocities, wall shear stress, intramural hematoma, thrombus in dissected false lumen, penetrating aortic ulcer or bicuspid aortic valve [56].

### Study Limitations

It was a retrospective single-center analysis of MRA in a cohort of European consecutive patients both females and males, without previously diagnosed aortic pathology. Our results were based on a group of patients who underwent diagnostics due to non-vascular pathology, but may be comparative to a general cardiovascular population. We aimed to analyze the risk of abnormal aortic diameters in patients burdened with a high cardiovascular risk profile. Further studies are required to compare our results with larger-scale populations, including those without any cardiovascular risk factors.

The strength of our study is related to its adherence to the screening and prevention programs. Despite the evidence of lifestyle significance in the prevention of cardiovascular diseases, the prevalence of smoking, obesity and diabetes is high, and the patients’ adherence to pharmacotherapy of lipid disorders and arterial hypertension is still inadequate. Our study emphasizes the necessity of broadening the diagnostics of aortic pathology in medium-age patients with coronary artery disease, to enhance the recognition of aortic dilatation and reduce the risk of acute aortic syndromes.

## 5. Conclusions

The results of our analysis, identify that both imaging and clinical parameters reflect the relationship between the ascending aorta and the thoracic and abdominal aorta dilatation. Patients with ascending aorta dilatation and co-existence of coronary artery disease, particularly those over the age of 49 or 54 years, respectively, are more prone to thoracic and abdominal aorta dilatation.

## Data Availability

The data supporting their findings may be obtained from the corresponding authors after reasonable explanation of requirement by e-mail contact for 3 years following the publications.

## References

[1] Chaikof EL, Dalman RL, Eskandari MK, Jackson BM, Lee WA, Mansour MA (2018). The Society for Vascular Surgery practice guidelines on the care of patients with an abdominal aortic aneurysm. *Journal of Vascular Surgery*.

[2] Senser EM, Misra S, Henkin S (2021). Thoracic Aortic Aneurysm: A Clinical Review. *Cardiology Clinics*.

[3] Schmitz-Rixen T, Keese M, Hakimi M, Peters A, Böckler D, Nelson K (2016). Ruptured abdominal aortic aneurysm-epidemiology, predisposing factors, and biology. *Langenbeck’s Archives of Surgery*.

[4] Ganapathi AM, Ranney DN, Peterson MD, Lindsay ME, Patel HJ, Pyeritz RE (2022). Location of Aortic Enlargement and Risk of Type A Dissection at Smaller Diameters. *Journal of the American College of Cardiology*.

[5] Quintana RA, Taylor WR (2019). Cellular Mechanisms of Aortic Aneurysm Formation. *Circulation Research*.

[6] Guo MH, Appoo JJ, Saczkowski R, Smith HN, Ouzounian M, Gregory AJ (2018). Association of Mortality and Acute Aortic Events with Ascending Aortic Aneurysm: A Systematic Review and Meta-analysis. *JAMA Network Open*.

[7] Jauhiainen S, Kiema M, Hedman M, Laakkonen JP (2022). Large Vessel Cell Heterogeneity and Plasticity: Focus in Aortic Aneurysms. *Arteriosclerosis, Thrombosis, and Vascular Biology*.

[8] Kiema M, Sarin JK, Kauhanen SP, Torniainen J, Matikka H, Luoto ES (2022). Wall Shear Stress Predicts Media Degeneration and Biomechanical Changes in Thoracic Aorta. *Frontiers in Physiology*.

[9] Li Z, Cong X, Kong W (2022). Matricellular proteins: Potential biomarkers and mechanistic factors in aortic aneurysms. *Journal of Molecular and Cellular Cardiology*.

[10] Tijmes FS, Karur GR (2022). Imaging of Heritable Thoracic Aortic Disease. *Seminars in Roentgenology*.

[11] Schanzer A, Oderich GS (2021). Management of Abdominal Aortic Aneurysms. *The New England Journal of Medicine*.

[12] Watson JDB, Gifford SM, Bandyk DF (2020). Aortic aneurysm screening using duplex ultrasound: Choosing wisely who to examine. *Seminars in Vascular Surgery*.

[13] Mantella LE, Chan W, Bisleri G, Hassan SMA, Liblik K, Benbarkat H (2020). The use of ultrasound to assess aortic biomechanics: Implications for aneurysm and dissection. *Echocardiography (Mount Kisco, N.Y.)*.

[14] Sprynger M, Willems M, Van Damme H, Drieghe B, Wautrecht JC, Moonen M (2019). Screening Program of Abdominal Aortic Aneurysm. *Angiology*.

[15] Benson RA, Meecham L, Fisher O, Loftus IM (2018). Ultrasound screening for abdominal aortic aneurysm: current practice, challenges and controversies. *The British Journal of Radiology*.

[16] Adriaans BP, Wildberger JE, Westenberg JJM, Lamb HJ, Schalla S (2019). Predictive imaging for thoracic aortic dissection and rupture: moving beyond diameters. *European Radiology*.

[17] Bossone E, Eagle KA (2021). Epidemiology and management of aortic disease: aortic aneurysms and acute aortic syndromes. *Nature Reviews. Cardiology*.

[18] Golledge J (2019). Abdominal aortic aneurysm: update on pathogenesis and medical treatments. *Nature Reviews. Cardiology*.

[19] Saeyeldin AA, Velasquez CA, Mahmood SUB, Brownstein AJ, Zafar MA, Ziganshin BA (2019). Thoracic aortic aneurysm: unlocking the “silent killer” secrets. *General Thoracic and Cardiovascular Surgery*.

[20] van Hout MJ, Scholte AJ, Juffermans JF, Westenberg JJ, Zhong L, Zhou X (2020). How to Measure the Aorta Using MRI: A Practical Guide. *Journal of Magnetic Resonance Imaging: JMRI*.

[21] Chen T, Yang X, Fang X, Tang L, Zhang Y, Weng Y (2022). Potential influencing factors of aortic diameter at specific segments in population with cardiovascular risk. *BMC Cardiovascular Disorders*.

[22] Chen X, Peng F, Liu X, Xia J, Niu H, He X (2023). Three-dimensional aneurysm wall enhancement in fusiform intracranial aneurysms is associated with aneurysmal symptoms. *Frontiers in Neuroscience*.

[23] Garcier JM, Petitcolin V, Filaire M, Mofid R, Azarnouch K, Ravel A (2003). Normal diameter of the thoracic aorta in adults: a magnetic resonance imaging study. *Surgical and Radiologic Anatomy: SRA*.

[24] Di Cesare E, Splendiani A, Barile A, Squillaci E, Di Cesare A, Brunese L (2016). CT and MR imaging of the thoracic aorta. *Open Medicine (Warsaw, Poland)*.

[25] Obel LM, Diederichsen AC, Steffensen FH, Frost L, Lambrechtsen J, Busk M (2021). Population-Based Risk Factors for Ascending, Arch, Descending, and Abdominal Aortic Dilations for 60-74-Year-Old Individuals. *Journal of the American College of Cardiology*.

[26] Ehrman JK, Fernandez AB, Myers J, Oh P, Thompson PD, Keteyian SJ (2020). Aortic Aneurysm: diagnosis, management, exercise testing, and training. *Journal of Cardiopulmonary Rehabilitation and Prevention*.

[27] Isselbacher EM (2005). Thoracic and abdominal aortic aneurysms. *Circulation*.

[28] Altobelli E, Rapacchietta L, Profeta VF, Fagnano R (2018). Risk Factors for Abdominal Aortic Aneurysm in Population-Based Studies: A Systematic Review and Meta-Analysis. *International Journal of Environmental Research and Public Health*.

[29] Goodney PP, Wang G (2022). Improving Screening for Aortic Aneurysm with Data Science. *JAMA*.

[30] Kobeissi E, Hibino M, Pan H, Aune D (2019). Blood pressure, hypertension and the risk of abdominal aortic aneurysms: a systematic review and meta-analysis of cohort studies. *European Journal of Epidemiology*.

[31] Shuai T, Kan Y, Si Y, Fu W (2020). High-risk factors related to the occurrence and development of abdominal aortic aneurysm. *Journal of Interventional Medicine*.

[32] Wilmink TB, Quick CR, Day NE (1999). The association between cigarette smoking and abdominal aortic aneurysms. *Journal of Vascular Surgery*.

[33] Prakash SK, Pedroza C, Khalil YA, Milewicz DM (2012). Diabetes and reduced risk for thoracic aortic aneurysms and dissections: a nationwide case-control study. *Journal of the American Heart Association*.

[34] Shantikumar S, Ajjan R, Porter KE, Scott DJA (2010). Diabetes and the abdominal aortic aneurysm. *European Journal of Vascular and Endovascular Surgery: the Official Journal of the European Society for Vascular Surgery*.

[35] Wang L, Djousse L, Song Y, Akinkuolie AO, Matsumoto C, Manson JE (2017). Associations of Diabetes and Obesity with Risk of Abdominal Aortic Aneurysm in Men. *Journal of Obesity*.

[36] Ballegaard CR, Pham MHC, Sigvardsen PE, Kühl JT, Sørgaard M, Taudorf M (2022). Aortic enlargement and coronary artery calcification in a general population cohort. *European Heart Journal. Cardiovascular Imaging*.

[37] Hernesniemi JA, Vänni V, Hakala T (2015). The prevalence of abdominal aortic aneurysm is consistently high among patients with coronary artery disease. *Journal of Vascular Surgery*.

[38] Palazzuoli A, Gallotta M, Guerrieri G, Quatrini I, Franci B, Campagna MS (2008). Prevalence of risk factors, coronary and systemic atherosclerosis in abdominal aortic aneurysm: comparison with high cardiovascular risk population. *Vascular Health and Risk Management*.

[39] Kim H, Kim J, Choe YH, Kim SM (2023). The Prognostic Impact of Coronary Artery Disease and Aortic Aneurysm: Insights from CT Protocol for Simultaneous Evaluation of Coronary Artery and Aorta. *Journal of Korean Medical Science*.

[40] Evangelista A, Flachskampf FA, Erbel R, Antonini-Canterin F, Vlachopoulos C, Rocchi G (2010). Echocardiography in aortic diseases: EAE recommendations for clinical practice. *European Journal of Echocardiography: the Journal of the Working Group on Echocardiography of the European Society of Cardiology*.

[41] Isekame Y, Gati S, Aragon-Martin JA, Bastiaenen R, Kondapally Seshasai SR, Child A (2016). Cardiovascular Management of Adults with Marfan Syndrome. *European Cardiology*.

[42] Srichai MB, Kim S, Axel L, Babb J, Hecht EM (2010). Non-gadolinium-enhanced 3-dimensional magnetic resonance angiography for the evaluation of thoracic aortic disease: a preliminary experience. *Texas Heart Institute Journal*.

[43] Erbel R, Aboyans V, Boileau C, Bossone E, Di Bartolomeo R, Eggebrecht H (2014). 2014 ESC Guidelines on the diagnosis and treatment of aortic diseases. *Kardiologia Polska*.

[44] Paul TK, Alamin AE, Subedi P, Alamian A, Wang L, Blackwell G (2021). Association Between Cardiovascular Risk Factors and the Diameter of the Thoracic Aorta in an Asymptomatic Population in the Central Appalachian Region. *The American Journal of the Medical Sciences*.

[45] Wang YD, Liu ZJ, Ren J, Xiang MX (2018). Pharmacological Therapy of Abdominal Aortic Aneurysm: An Update. *Current Vascular Pharmacology*.

[46] DeRoo E, Stranz A, Yang H, Hsieh M, Se C, Zhou T (2022). Endothelial Dysfunction in the Pathogenesis of Abdominal Aortic Aneurysm. *Biomolecules*.

[47] Milewicz DM, Trybus KM, Guo DC, Sweeney HL, Regalado E, Kamm K (2017). Altered Smooth Muscle Cell Force Generation as a Driver of Thoracic Aortic Aneurysms and Dissections. *Arteriosclerosis, Thrombosis, and Vascular Biology*.

[48] Xiang B, Zhu S, Li J, Lai H, Wang C, Zhu K (2021). Current Pharmacological Management of Aortic Aneurysm. *Journal of Cardiovascular Pharmacology*.

[49] Raffort J, Lareyre F, Clément M, Hassen-Khodja R, Chinetti G, Mallat Z (2018). Diabetes and aortic aneurysm: current state of the art. *Cardiovascular Research*.

[50] Su Z, Guo J, Gu Y (2022). Pharmacotherapy in Clinical Trials for Abdominal Aortic Aneurysms: A Systematic Review and Meta-Analysis. *Clinical and Applied Thrombosis/hemostasis: Official Journal of the International Academy of Clinical and Applied Thrombosis/Hemostasis*.

[51] Baman JR, Eskandari MK (2022). What Is an Abdominal Aortic Aneurysm. *JAMA*.

[52] Carrel T, Sundt TM, von Kodolitsch Y, Czerny M (2023). Acute aortic dissection. *The Lancet (London, England)*.

[53] Song XT, Fan L, Yan ZN, Rui YF (2021). Echocardiographic evaluation of the elasticity of the ascending aorta in patients with essential hypertension. *Journal of Clinical Ultrasound: JCU*.

[54] Song XT, Fan L, Yan ZN, Rui YF (2020). Evaluation of the Effect of Essential Hypertension on Elasticity of Ascending Aorta in Type 2 Diabetic Mellitus Patients by Echocardiography. *Angiology*.

[55] Gallego-Colon E, Yosefy C, Cherniavsky E, Osherov A, Khalameizer V, Piltz X (2021). Isolated ascending aorta dilatation is associated with increased risk of abdominal aortic aneurysm. *Journal of Cardiothoracic Surgery*.

[56] Erbel R, Aboyans V, Boileau C, Bossone E, Bartolomeo RD, Eggebrecht H (2014). 2014 ESC Guidelines on the diagnosis and treatment of aortic diseases: Document covering acute and chronic aortic diseases of the thoracic and abdominal aorta of the adult. The Task Force for the Diagnosis and Treatment of Aortic Diseases of the European Society of Cardiology (ESC). *European Heart Journal*.

